# The noninferiority of transcatheter aortic valve implantation compared to surgical aortic valve replacement for severe aortic disease: Evidence based on 16 randomized controlled trials

**DOI:** 10.1097/MD.0000000000026556

**Published:** 2021-07-16

**Authors:** Peng-Ying Zhao, Yong-Hong Wang, Rui-Sheng Liu, Ji-Hai Zhu, Jian-Ying Wu, Bing Song

**Affiliations:** a Department of Cardiovascular Surgery, Lanzhou University First Affiliated Hospital, Lanzhou, China; b Department of Cardiovascular Surgery, Qinghai University Affiliated Hospital, Xi’ning, China; c Medical College of Qinghai University, Qinghai University, Xi’ning, China.

**Keywords:** aortic valve replacement, mortality, noninferiority, superiority, transcatheter aortic valve implantation

## Abstract

**Background:**

Currently, transcatheter aortic valve implantation (TAVI) as an effective and convenient intervention has been adopted extensively for patients with severe aortic disease. However, the efficacy and safety of TAVI have not yet been well evaluated and its noninferiority compared with traditional surgical aortic valve replacement (sAVR) still lack sufficient evidence. This meta-analysis was designed to comprehensively compare the noninferiority of TAVI with sAVR for patients with severe aortic disease.

**Methods:**

A systematic search of PubMed, Embase, Cochrane Library, and Web of Science up to October 1, 2020 was conducted for relevant studies that comparing TAVI and sAVR in the treatment of severe aortic disease. The primary outcomes were early, midterm and long term mortality. The secondary outcomes included early complications and other late outcomes. Two reviewers assessed trial quality and extracted the data independently. All statistical analyzes were performed using the standard statistical procedures provided in Review Manager 5.2.

**Results:**

A total of 16 studies including 14394 patients were identified. There was no difference in 30-day, 1-year, 2-year, and 5-year all-cause or cardiovascular mortality as well as stroke between TAVI and sAVR. Regarding to the 30-day outcomes, compared with sAVR, TAVI experienced a significantly lower incidence of myocardial infarction (risk ratio [RR] 0.62; 95% confidence interval [CI] 0.40–0.97; 5441 pts), cardiogenic shock (RR 0.34; 95% CI 0.19–0.59; 1936 pts), acute kidney injury (AKI) > stage 2 (RR 0.37; 95% CI 0.25–0.54; 5371 pts), and new-onset atrial fibrillation (NOAF) (RR 0.29; 95% CI 0.24–0.35; 5371 pts) respectively, but higher incidence of permanent pacemaker implantation (RR 3.16; 95% CI 1.61–6.21; 5441 pts) and major vascular complications (RR 2.22; 95% CI 1.14–4.32; 5371 pts). Regarding to the 1- and 2-year outcomes, compared with sAVR, TAVI experienced a significantly lower incidence of NOAF, but higher incidence of neurological events, transient ischemic attacks (TIA), permanent pacemaker and major vascular complications respectively. Regarding to the 5-year outcomes, compared with sAVR, TAVI experienced a significantly lower incidence of NOAF, but higher incidence of TIA and reintervention respectively.

**Conclusions:**

Our analysis shows that TAVI was equal to sAVR in early, midterm and long term mortality for patients with severe aortic disease. In addition, TAVI may be favorable in reducing the incidence of both early, midterm and long term NOAF. However, pooled results showed superiority of sAVR in reducing permanent pacemaker implantation, neurological events, TIA, major vascular complications and reintervention.

## 1 Introduction

At present, degenerative aortic valve disease, as one of the most frequent valvular heart disease with a severity ranging from aortic sclerosis slowly progressing to symptomatic severe aortic stenosis (AS), usually requires aortic valve replacement.^[1]^ In patients older than 75 years, AS is present in 12.4% of the population, with severe forms in 3.4% of the elderly.^[2]^ Currently, though surgical aortic valve replacement (sAVR) was a traditional effective method for patients with symptomatic severe AS, transcatheter aortic valve implantation (TAVI) as an effective and convenient intervention has been adopted extensively.

According to the European and American guidelines, symptomatic severe AS requires sAVR or TAVI, with a mean survival of 2 to 3 years in the absence of these procedures.^[3,4]^ TAVI is increasingly used in high and more recently in intermediate-risk population, studies evaluating now the indication even in low-risk population.^[5–8]^ The 2017 American Heart Association Valvular Guidelines^[9,10]^ have given TAVR a Class I recommendation (level of evidence A) for these patients at high or prohibitive surgical risk. For those at intermediate risk, TAVR is considered a reasonable alternative to SAVR,^[7,11]^ with a Class IIA recommendation in the American Heart Association guidelines.^[9,10]^ These decisions should involve a multi-disciplinary heart valve team.

However, the efficacy and safety of TAVI have not yet been well evaluated and its noninferiority compared with traditional sAVR still lack sufficient evidence. In addition, the long term outcomes between TAVI and sAVR have not yet be compared at present his meta-analysis was designed to comprehensively compare the early, midterm and long term noninferiority and superiority of TAVI with sAVR for patients with severe aortic disease.

## 2 Methods

### 2.1 Search strategy and study selection

A systematic search of PubMed, Embase, Cochrane Library, and Web of Science up to October 1, 2020 was conducted for relevant studies using a search strategy developed by a medical information specialist that involved controlled vocabulary and keywords related to our research question (e.g., “aortic stenosis,” “valvular heart disease,” “aortic valve disease”; “transcatheter aortic valve replacement,” “transcatheter aortic valve implantation,” “surgical aortic valve replacement,” “surgical aortic valve implantation,” “TAVR,” “TAVI,” “SAVR,” “SAVI”; “survival,” “outcome” “prognosis,” “mortality,” “complication”). The search strategy was limited to English language articles. Two assessors independently screened the titles and abstracts of each study. When a relevant study was identified, its full text was obtained for further evaluation. The full text of related references was also obtained for review.

The present study was approved by the Ethics Committee of Lanzhou University First Affiliated Hospital.

### 2.2 Criteria for considering studies

We included studies if they met the following criteria: a. randomized controlled trials (RCTs) that compared TAVI with sAVR; b. studies in which the relevant outcomes of both TAVI and sAVR groups were assessed; and c. patients who were diagnosed with severe aortic disease.

Studies were excluded if they met the following criteria: a. experimental trial on animals or a non-human study, non-RCTs, quasi-RCTs, or observational studies; b. study population included patients with other diseases that would affect outcomes; c. study reported in the form of an abstract, letter, editorial, expert opinion, review, or case report; or d. lack of sufficient data or failure to meet the inclusion criteria.

### 2.3 Quality assessment and data extraction

Two reviewers independently assessed the quality of each RCT using the previously validated 5-point Jadad scale,^[12]^ and disagreement was resolved by their discussion. Studies with scores of 0 to 1 were considered low quality; scores of 2 to 3 were considered moderate quality; scores of 4 or more were considered high quality. In addition, the risk of bias for each studies and the risk of bias across all studies were evaluated and shown with figures generated by RevMan 5.2 software.^[13]^

Baseline characteristics and outcomes from the included studies were extracted using a standardized extraction form. Key study characteristics including study year, sample size, sex, mean age, intervention, follow-up time and outcomes, were extracted. Data were extracted by 1 reviewer and then examined for accuracy and completeness by a second reviewer. The disagreement was resolved by their discussion.

### 2.4 Outcome measures

The primary outcomes were early, midterm and long term mortality.

The secondary outcomes included early complications and other late outcomes.

### 2.5 Data synthesis and statistical methods

The data of comparable outcomes between TAVI and sAVR were combined-analyzed, using the standard statistical procedures provided in RevMan 5.2.^[13]^ Dichotomous data were measured with risk ratio (RR) and continuous variable data were measured with mean difference (MD). The heterogeneity between studies was evaluated by the Chi-Squared based Q statistical test,^[14]^ with *P*_*h*_ value and *I*^*2*^ statistic, ranging from 0% to 100%, to quantify the effect of heterogeneity. *P*_*h*_ ≤ 0.10 was deemed to represent significant heterogeneity,^[15]^ and pooled estimates were estimated using a random-effect model (the DerSimonian and Laird method^[16]^). On the contrary, if statistical study heterogeneity was not observed (*P*_*h*_ > 0.10), a fixed effects model (the Mantel–Haenszel method^[17]^) was used. The effects of outcome measures were considered to be statistically significant if pooled RRs with 95% CI did not overlap with 1 or pooled MDs with 95% CI did not overlap with 0.

This work has been reported in line with Preferred Reporting Items for Systematic Reviews and Meta-Analyzes^[18]^ and Assessing the methodological quality of systematic reviews Guidelines.^[19]^

## 3 Results

### 3.1 Included studies, study characteristics, and quality assessment

**Figure 1:**
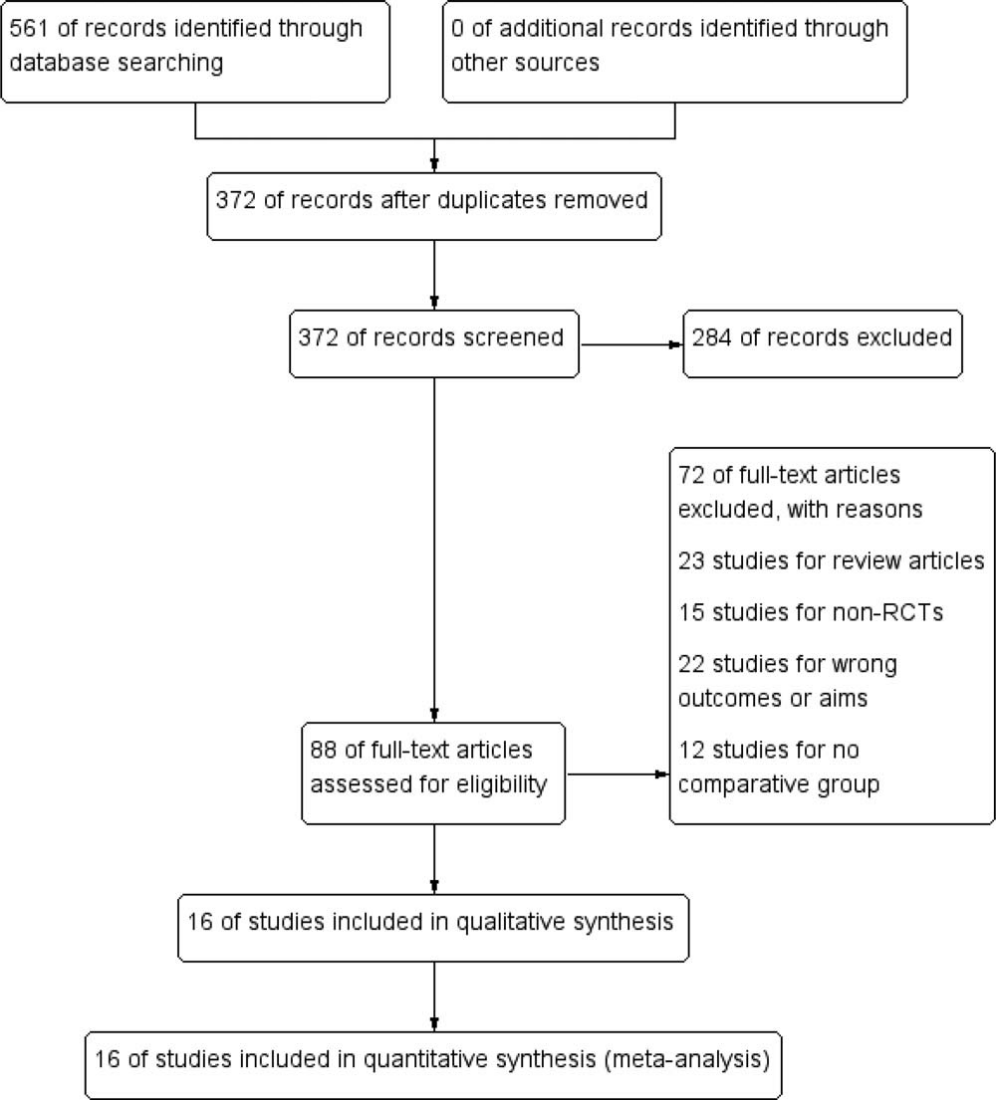
Flow diagram of literature search and selection of included studies for meta-analysis. At the beginning, a total of 561 records of citations were obtained; 372 of records were reviewed further after duplicates were removed. Via screening the titles and abstracts, 129 studies were excluded preliminarily and then 88 studies were chosen to get full texts for further evaluation. After reading the full texts, 72 studies were excluded further (23 studies for review articles, 15 for non-RCTs, 12 for lack of controls and 22 for erroneous aims). Eventually, 16 RCTs were included for meta-analysis.

At the beginning of the search, a total of 561 records of citations were obtained; 372 of records were reviewed further after duplicates were removed. Via screening the titles and abstracts, 129 studies were excluded preliminarily and then 88 studies were chosen to get full texts for further evaluation. After reading the full texts, 72 studies were excluded further (23 studies for review articles, 15 for non-RCTs, 12 for lack of controls and 22 for erroneous aims). Eventually, 16 RCTs^[7,8,11,20–32]^ (N = 14394 participants) were included in this systematic review and meta-analysis. Of these studies, except one study,^[23]^ the others were about multicenter studies. The detailed search process and summary of studies are shown in the study flow diagram (Fig. 1). The other characteristics of each study are shown in Table 1.

**Table 1:** The characteristics of included RCTs for meta-analysis.

		Sample size		Female (n)							
Study	Year	TAVI	sAVR	TAVI	sAVR	Age (mean ± SD, year)	STS score (mean ± SD, %)	Location	Follow-up time	Primary outcomes	Jadad score
Kapadia SR et al	2015	179	179	193		NR	11.7	Multicenter	60 mo	All-cause mortality at 1 yr, cardiovascular mortality, stroke, vascular complications, major bleeding, and functional status.	4
Leon MB et al	2016	1011	1021	463	461	81.5 ± 6.7 81.7 ± 6.7	5.8 ± 2.1 5.8 ± 1.9	Multicenter	24 mo	Death from any cause or disabling stroke at 2 yr	5
Mack MJ et al	2015	348	351	300		84.1 ± 6.6	11.8 ± 3.3 11.7 ± 3.5	Multicenter	60 mo	All-cause mortality in the ITT population at 1 and 5 yr,	4
Mack MJ et al	2019	496	454	161	131	73.3 ± 5.8 73.6 ± 6.1	1.9 ± 0.7 1.9 ± 0.6	Multicenter	12 mo	Composite of all-cause death, stroke, or rehospitalization at 1 yr	4
Makkar RR et al	2020	994	994	446	434	81.5 ± 6.7 81.7 ± 6.7	5.8 ± 2.1 5.8 ± 1.9	Multicenter	60 mo	Nonhierarchical composite of death from any cause or disabling stroke at 2 yr in the ITT population	4
Miller DC et al	2012	344	313	146	134	83.6 ± 6.8 84.4 ± 6.3	11.8 ± 3.3 11.7 ± 3.4	NR	24 mo	All neurologic events and all-cause mortality	4
Nielsen HH et al	2012	34	36	25	24	80 ± 3.6 82 ± 4.4	3.1 ± 1.5 3.4 ± 1.2	Multicenter	3 mo	The composite of all-cause mortality, cerebral stroke and/or RF requiring haemodialysis at 30 d	4
Pibarot P et al	2020	495	453	NR	NR	NR	NR	Multicenter	NR	Transthoracic echocardiograms obtained at baseline, and at 30 d and 1 yr post-procedure were analyzed	3
Popma JJ et al	2019	725	678	261	229	74.1 ± 5.8 73.6 ± 5.9	1.9 ± 0.7 1.9 ± 0.7	Multicenter	12.2 mo	Composite of all-cause death or disabling stroke at 24 mo	4
Reardon MJ et al	2015	391	359	184	171	83.2 ± 7.1 83.3 ± 6.3	7.3 ± 3.0 7.5 ± 3.3	Multicenter	24.4 mo	The 2-yr clinical and echocardiographic outcomes	4
Reardon MJ et al	2016	202	181	85	80	81.5 ± 7.6 81.2 ± 6.6	5.3 (4.3–6.1) 5.3 (4.1–5.9)	Multicenter	24 mo	All-cause mortality and quality of life through 2 yr	4
Reardon MJ et al	2017	864	796	366	358	79.9 ± 6.2 79.7 ± 6.1	4.4 ± 1.5 4.5 ± 1.6	Multicenter	24 mo	Composite of death from any cause or disabling stroke at 24 mo	5
Serruys PW et al	2018	1660		724		75.1 ± 6.5 75.4 ± 5.5 80.0 ± 5.7 79.9 ± 5.7 82.3 ± 5.6 81.4 ± 6.0	2.3 ± 0.5 2.3 ± 0.5 4.0 ± 0.6 4.0 ± 0.6 6.2 ± 1.0 6.3 ± 1.1	Multicenter	24 mo	Composite of all-cause death or disabling stroke at 24 mo	4
Søndergaard L et al	2016	142	134	66	64	79.2 ± 4.9 79.0 ± 4.7	2.9 ± 1.6 3.1 ± 1.7	Multicenter	24 mo	The composite rate of death from any cause, stroke, or MI	4
Thyregod HG et al	2015	145	135	67	64	79.2 ± 4.9 79.0 ± 4.7	2.9 3.1	Multicenter	12 mo	The composite rate of death from any cause, stroke, or MI at 1 yr	4
Thyregod HGH et al	2019	280		78	71	79.1 ± 4.8	3.0 ± 1.7	Multicenter	60 mo	The rate of all-cause mortality, stroke, or MI	4

ITT = intention-to-treat, MI = myocardial infarction, RF = renal failure, sAVR = surgical aortic valve replacement, SD = standard deviation, STS score = the Society of Thoracic Surgeons score, TAVI = transcatheter aortic valve implantation.

**Figure 2:**
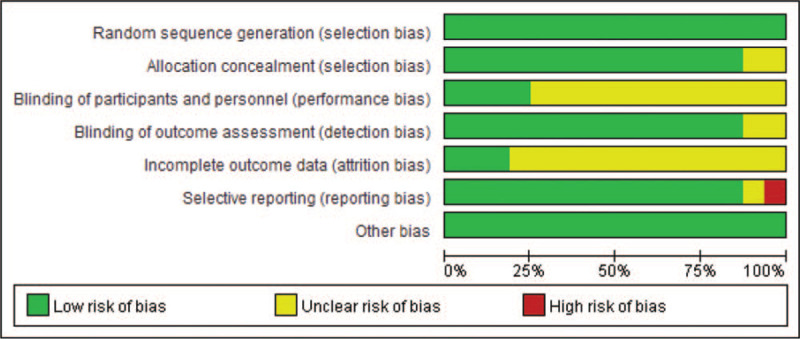
Risk of bias graph: review authors’ judgements about each risk of bias item presented as percentages across all included studies.

**Figure 3:**
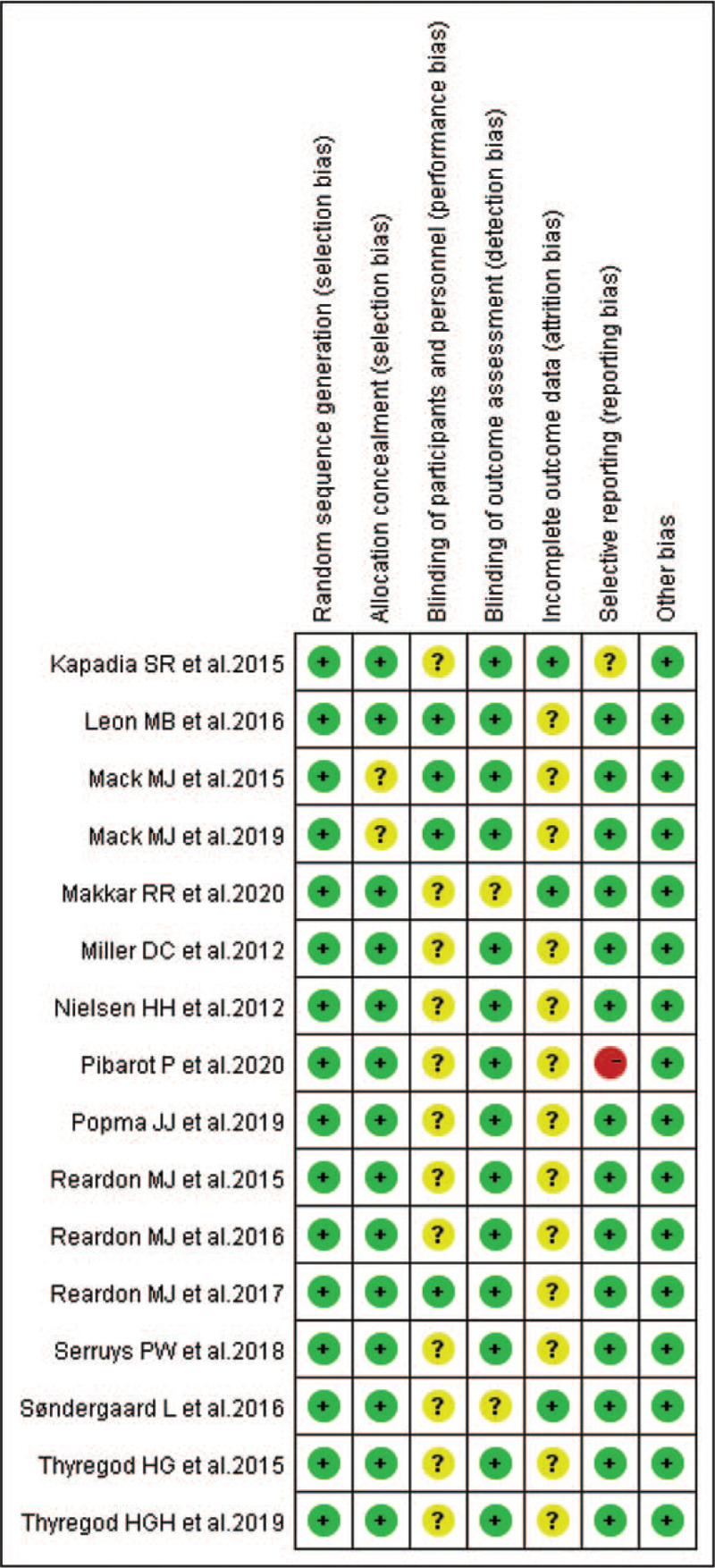
Risk of bias summary: review authors’ judgements about each risk of bias item for each included study.

According to our definitions, there was no low quality studies included in this analysis. Except Pibarot et al (2020)^[25]^ evaluated as moderate quality, the other studies were rated as high quality (93.7%). Additionally, risk-of-bias graphs were generated to further identify the risk of bias of the including studies. The risk of bias for each RCT was presented as percentages across all included studies, and the risk-of-bias item for each included study was displayed (Figs. 2 and 3). The risk-of-bias graphs indicated generally low risk of selection, detection, reporting and other bias. All studies experienced low risk of bias in “Random sequence generation” item and other bias. A high risk of bias was mainly observed in reporting bias in one study.^[25]^ An unclear risk of bias was mainly observed in performance and attrition bias.

### 3.2 Comparison between transcatheter aortic valve implantation and surgical aortic valve replacement regarding to baseline characteristics

We compared the baseline characteristics of both TAVI and sAVR groups with a total of 16 studies (N = 14394 participants). As Table 2 showing, there was no difference between TAVI and sAVR groups in age (MD −0.06; 95% confidence interval [CI] −0.30–0.18; 10423 pts), left ventricular ejection fraction (LVEF) (%) (MD −0.39; 95% CI −0.94–0.15; 3986 pts), aortic valve area (cm^2^) (MD 0.02; 95% CI −0.04–0.07; 3080 pts), and aortic-valve peak gradient (mm Hg) (MD 0.64; 95% CI −1.11–2.38; 3080 pts) respectively. In addition, there was also no difference between TAVI and sAVR groups in the proportion of diabetes mellitus, serum creatinine >2 mg/dL, prior stroke, prior transient ischemic attacks (TIA), peripheral vascular disease, prior pacemaker implantation, prior coronary-artery bypass grafting, prior percutaneous coronary intervention, prior myocardial infarction (MI), history of arrhythmia, atrial fibrillation, NYHA Class III/IV, cerebral vascular disease, chronic obstructive pulmonary disease, pulmonary hypertension and hypertension respectively. However, significant difference between TAVI and sAVR groups was observed in the proportion of coronary artery disease (CAD) (RR 0.96; 95% CI 0.92–1.0; 5671 pts) and congestive heart failure (MD 0.98; 95% CI 0.97–1.00; 3320 pts).

**Table 2:** The pooled baseline characteristics results of comparison between TAVI and sAVR for severe AS.

		Pooled results			Heterogeneity		
Subgroups	No. of study/pts	MD/RR	95% CI	*P* value	*I*^2^	*P*_*h*_ value	Analytical effect model
Age	11/10423	MD −0.06	−0.30, 0.18	.61	13%	.32	Fixed effects model
DM	7/6772	RR 0.96	0.90, 1.03	.25	29%	.21	Fixed effects model
Serum Cr > 2 mg/dl	6/6022	RR 0.88	0.56, 1.38	.57	0%	.80	Fixed effects model
Prior stroke	5/5058	RR 0.88	0.72, 1.07	.20	0%	.86	Fixed effects model
Prior TIA	4/4718	RR 1.09	0.88, 1.34	.44	0%	.86	Fixed effects model
PVD	8/7405	RR 1.0	0.93, 1.08	1.00	0%	.97	Fixed effects model
Prior PM	5/7354	RR 1.0	0.87, 1.14	.97	0%	.92	Fixed effects model
CAD	5/5671	RR 0.96	0.92, 1.0	.04	16%	.31	Fixed effects model
Prior CABG	5/6124	RR 0.94	0.85, 1.04	.25	0%	.97	Fixed effects model
Prior PCI	6/6395	RR 1.0	0.91, 1.09	.99	0%	.89	Fixed effects model
Prior MI	6/6700	RR 1.06	0.93, 1.20	.40	0%	.88	Fixed effects model
CHF	2/3320	RR 0.98	0.97, 1.00	.02	0%	.64	Fixed effects model
History of arrhythmia	2/3320	RR 1.01	0.92, 1.12	.79	0%	1.0	Fixed effects model
AF	7/7271	RR 0.96	0.89, 1.04	.32	2%	.41	Fixed effects model
NYHA Class III/IV	7/7358	RR 1.01	0.96, 1.06	.66	50%	.06	Random-effect model
CVD	4/2358	RR 0.97	0.81, 1.17	.78	0%	.76	Fixed effects model
COPD	5/3092	RR 0.91	0.80, 1.03	.14	0%	.74	Fixed effects model
LVEF (%)	5/3986	MD −0.39	−0.94, 0.15	.16	0%	.95	Fixed effects model
Aortic valve area (cm^2^)	4/3080	MD 0.02	−0.04, 0.07	.51	91%	<.0001	Random-effect model
Aortic-valve peak gradient (mm Hg)	4/3080	MD 0.64	−1.11, 2.38	.48	63%	.05	Random-effect model
PH	2/1563	RR 1.02	0.88, 1.19	.76	0%	.54	Fixed effects model
Hypertension	4/4091	RR 1.01	0.99, 1.04	.23	20%	.36	Fixed effects model

AF = atrial fibrillation, CABG = coronary-artery bypass grafting, CAD = coronary artery disease, CHF = congestive heart failure, CI = confidence intervals, COPD = chronic obstructive pulmonary disease, Cr = creatinine, CVD = cerebral vascular disease, DM = diabetes mellitus, LVEF = left ventricular ejection fraction, MD = mean difference, MI = myocardial infarction, PCI = percutaneous coronary intervention, PH = pulmonary hypertension, PM = pacemaker, PVD = peripheral vascular disease, RR = risk ratio, TIA = transient ischemic attacks.

### 3.3 Comparison between transcatheter aortic valve implantation and surgical aortic valve replacement regarding to the 30-day outcomes

**Figure 4:**
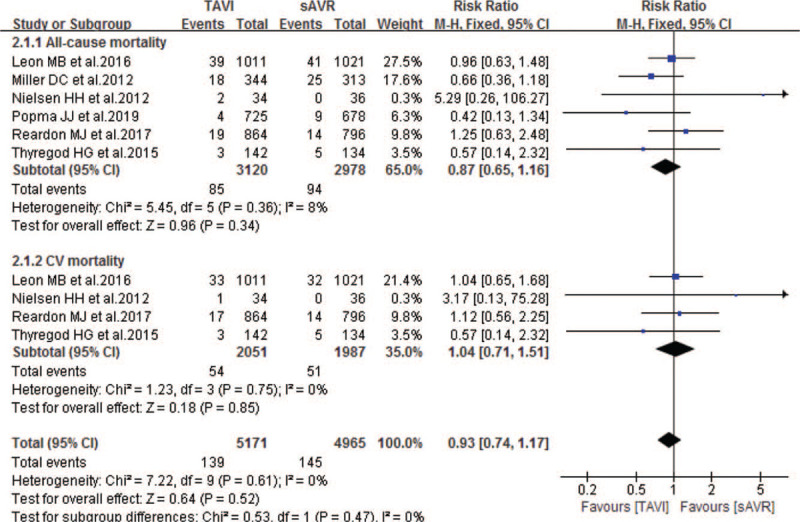
Forest plot of comparison between TAVI and sAVR for severe AS regarding to 30-day mortality.

Six studies compared 30-day mortality of patients with severe AS between TAVI and sAVR groups. As Fig. 4 showing, pooled results showed no significant difference in the incidence of 30-day all-cause and CV mortality between TAVI and sAVR groups, with pooled RRs of 0.87 (95% CI 0.65–1.16; *P* = .34; 6098 pts) and 1.04 (95% CI 0.71–1.51; *P* = .85; 4038 pts) respectively. Similarly, compared with sAVR, TAVI showed non-inferiority in the following 30-day outcomes: stroke, TIA, life-threatening bleeding, neurological events, endocarditis, CAD, reintervention and rehospitalization (Table 3). In addition, one study also showed noninferiority between TAVI and sAVR in 30-day leakage, cardiac perforation and LVEF. However, compared with sAVR, TAVI experienced a significantly lower incidence of myocardial infarction (MI) (RR 0.62; 95% CI 0.40–0.97; 5441 pts), cardiogenic shock (RR 0.34; 95% CI 0.19–0.59; 1936 pts), AKI > stage 2 (RR 0.37; 95% CI 0.25–0.54; 5371 pts), and new-onset atrial fibrillation (NOAF) (RR 0.29; 95% CI 0.24–0.35; 5371 pts), but higher incidence of permanent pacemaker implantation (RR 3.16; 95% CI 1.61–6.21; 5441 pts) and major vascular complications (RR 2.22; 95% CI 1.14–4.32; 5371 pts) respectively (Table 3).

**Table 3:** The pooled results of comparison between TAVI and sAVR for severe AS regarding to the 30-day outcomes.

		Pooled results			Heterogeneity		
Subgroups	No. of study/pts	MD/RR	95% CI	*P* value	*I*^2^	*P*_h_ value	Analytical effect model
All-cause mortality	6/6098	RR 0.87	0.65, 1.16	.34	8%	.36	Fixed effects model
CV mortality	4/4038	RR 1.04	0.71, 1.51	.85	0%	.75	Fixed effects model
Stroke	5/5441	RR 0.82	0.64, 1.04	.10	0%	.42	Fixed effects model
TIA	5/5441	RR 1.50	0.85, 2.66	.16	0%	.66	Fixed effects model
MI	5/5441	RR 0.62	0.40, 0.97	.04	0%	.79	Fixed effects model
Bleeding	5/5441	RR 0.51	0.20, 1.28	.15	96%	<.0001	Random-effect model
Leakage	1/70	RR 2.12	0.41, 10.82	.37			
Permanent PM	5/5441	RR 3.16	1.61, 6.21	.0008	90%	<.0001	Random-effect model
Cardiogenic shock	2/1936	RR 0.34	0.19, 0.59	.002	0%	.64	Fixed effects model
Major vascular complications	4/5371	RR 2.22	1.14, 4.32	.02	77%	.004	Random-effect model
AKI > 2	4/5371	RR 0.37	0.25, 0.54	<.0001	0%	.64	Fixed effects model
Neurological events	2/2308	RR 0.99	0.72, 1.37	.96	0%	.94	Fixed effects model
Endocarditis	3/3711	RR 1.57	0.21, 11.80	.66	0%	.61	Fixed effects model
NOAF	4/5371	RR 0.29	0.24, 0.35	<.0001	56%	.08	Random-effect model
CAD	3/5095	RR 1.37	0.60, 3.16	.45	13%	.32	Fixed effects model
Reintervention	3/5095	RR 2.66	1.01, 7.00	.05	20%	.29	Fixed effects model
Rehospitalization	3/5095	RR 0.85	0.66, 1.11	.24	46%	.16	Fixed effects model
Cardiac perforation	1/1660	RR 1.97	0.81, 4.82	.14			
LVEF	1/887	MD 0.20	−0.93, 1.33	.73			

AKI = acute kidney injury, CAD = coronary artery disease, CI = confidence intervals, CV = cardiovascular, LVEF = left ventricular ejection fraction, MD = mean difference, MI = myocardial infarction, NOAF = new-onset atrial fibrillation, PM = pacemaker, RR = risk ratio, TIA = transient ischemic attacks.

### 3.4 Comparison between transcatheter aortic valve implantation and surgical aortic valve replacement regarding to the 1-year outcomes

**Figure 5:**
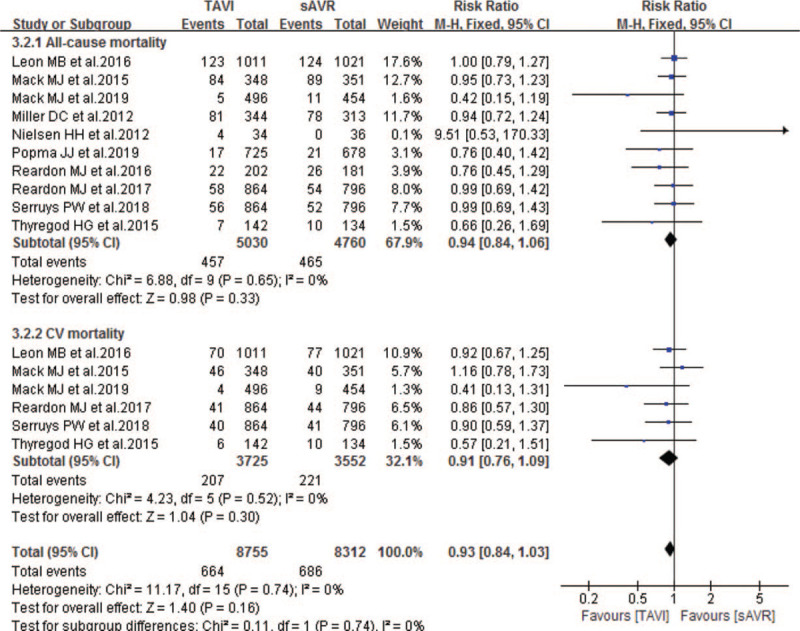
Forest plot of comparison between TAVI and sAVR for severe AS regarding to 1-year mortality.

Ten studies compared the 1-year mortality between TAVI and sAVR groups. As Fig. 5 showing, our pooled results also showed noninferiority in the incidence of 1-year all-cause and CV mortality of TAVI when compared to sAVR, with pooled RRs of 0.94 (95% CI 0.84–1.06; *P* = .33; 9790 pts) and 0.91 (95% CI 0.76–1.09; *P* = .30; 7277 pts) respectively. Similarly, compared with sAVR, TAVI showed noninferiority in the following 1-year outcomes: stroke, reintervention, MI, endocarditis, rehospitalization, aortic regurgitation and CAD (Table 4). In addition, one study also showed noninferiority between TAVI and sAVR in 1-year cardiac perforation, renal failure and LVEF. However, compared with sAVR, TAVI experienced a significantly lower incidence of life-threatening bleeding (RR 0.41; 95% CI 0.24–0.68; 6744 pts), all stage AKI (RR 0.44; 95% CI 0.25–0.77; 4642 pts), AKI > stage 2 (RR 0.56; 95% CI 0.40–0.77; 6045 pts), NOAF (RR 0.30; 95% CI 0.24–0.39; 6321 pts), but higher incidence of neurological events (RR 3.01; 95% CI 1.72–5.27; 6755 pts), TIA (RR 1.44; 95% CI 1.07–1.95; 8680 pts), major vascular complications (RR 2.23; 95% CI 1.19–4.18; 5794 pts) and permanent pacemaker implantation (RR 2.32; 95% CI 1.36–3.95; 7020 pts) respectively (Table 4).

**Table 4:** The pooled results of comparison between TAVI and sAVR for severe AS regarding to the 1-year outcomes.

		Pooled results			Heterogeneity		
Subgroups	No. of study/pts	MD/RR	95% CI	*P* value	*I*^2^	*P*_*h*_ value	Analytical effect model
All-cause mortality	10/9790	RR 0.94	0.84, 1.06	.33	0%	.65	Fixed effects model
CV mortality	6/7277	RR 0.91	0.76, 1.09	.30	0%	.52	Fixed effects model
Stroke	7/8680	RR 0.89	0.75, 1.06	.18	38%	.14	Fixed effects model
Neurological events	4/6755	RR 3.01	1.72, 5.27	.0001	0%	.46	Fixed effects model
Reintervention	3/3968	RR 0.96	0.78, 1.18	.67	0%	.42	Fixed effects model
TIA	7/8680	RR 1.44	1.07, 1.95	.02	0%	.88	Fixed effects model
Bleeding	5/6744	RR 0.41	0.24, 0.68	.0007	93%	<.0001	Random-effect model
Major vascular complications	4/5794	RR 2.23	1.19, 4.18	.01	83%	.0006	Random-effect model
All AKI	3/4642	RR 0.44	0.25, 0.77	.004	68%	.05	Random-effect model
AKI > stage 2	4/6045	RR 0.56	0.40, 0.77	.0004	49%	.12	Fixed effects model
MI	7/8680	RR 0.91	0.67, 1.23	.53	0%	.64	Fixed effects model
Cardiac perforation	1/1660	RR 2.15	0.83, 5.57	.11			
Cardiogenic shock	1/1660	RR 0.32	0.16, 0.65	.002			
Endocarditis	5/6070	RR 0.82	0.42, 1.58	.55	0%	.55	Fixed effects model
Rehospitalization	6/8404	RR 0.94	0.75, 1.18	.60	64%	.02	Random-effect model
Permanent PM	6/7020	RR 2.32	1.36, 3.95	.002	91%	<.0001	Random-effect model
NOAF	5/6321	RR 0.30	0.24, 0.39	<.0001	80%	.0005	Random-effect model
Aortic regurgitation	2/1852	RR 1.72	0.88, 3.34	.11	0%	.65	Fixed effects model
CAD	2/3435	RR 1.19	0.49, 2.88	.70	36%	.21	Fixed effects model
RF	1/699	RR 0.91	0.49, 1.69	.76			
LVEF	1/811	MD −0.10	−1.19, 0.99	.86			

AKI = acute kidney injury, CAD = coronary artery disease, CI = confidence intervals, CV = cardiovascular, LVEF = left ventricular ejection fraction, MD = mean difference, MI = myocardial infarction, NOAF = new-onset atrial fibrillation, PM = pacemaker, RF = renal failure, RR = risk ratio, TIA = transient ischemic attacks.

### 3.5 Comparison between transcatheter aortic valve implantation and surgical aortic valve replacement regarding to the 2-year outcomes

**Figure 6:**
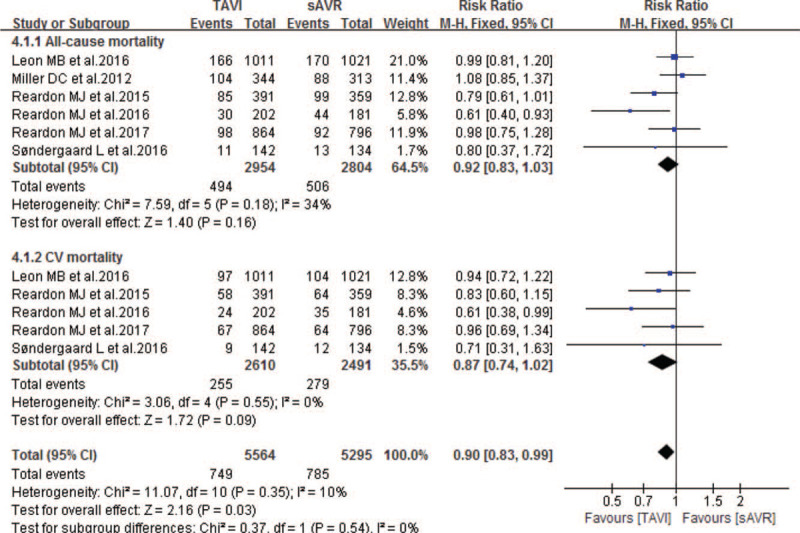
Forest plot of comparison between TAVI and sAVR for severe AS regarding to 2-year mortality.

Six studies compared the 2-year mortality between TAVI and sAVR groups. As Fig. 6 showing, our pooled results also showed noninferiority in the incidence of 2-year all-cause and CV mortality of TAVI when compared to sAVR, with pooled RRs of 0.92 (95% CI 0.83–1.03; *P* = .16; 5758 pts) and 0.87 (95% CI 0.74–1.02; *P* = .09; 5101 pts) respectively. Similarly, compared with sAVR, TAVI showed non-inferiority in the following 2-year outcomes: stroke, MI, life-threatening bleeding and all stage AKI (Table 5). In addition, one study also showed non-inferiority between TAVI and sAVR in 2-year endocarditis and CAD. However, compared with sAVR, TAVI experienced a significantly lower incidence of NOAF (RR 0.48; 95% CI 0.38–0.61; 3441 pts), but higher incidence of neurological events (RR 1.26; 95% CI 1.02–1.57; 2965 pts), TIA (RR 1.58; 95% CI 1.14–2.17; 5375 pts), permanent pacemaker implantation (RR 2.61; 95% CI 1.36–5.00; 3441 pts), rehospitalization (RR 1.25; 95% CI 1.06–1.46; 3692 pts), major vascular complications (RR 2.38; 95% CI 1.26–4.49; 3165 pts) and reintervention (RR 3.22; 95% CI 1.64–6.29; 3692 pts) respectively (Table 5).

**Table 5:** The pooled results of comparison between TAVI and sAVR for severe AS regarding to the 2-year outcomes.

		Pooled results			Heterogeneity		
Subgroups	No. of study/pts	RR	95% CI	*P* value	*I*^2^	*P*_*h*_ value	Analytical effect model
All-cause mortality	6/5758	0.92	0.83, 1.03	.16	34%	.18	Fixed effects model
CV mortality	5/5101	0.87	0.74, 1.02	.09	0%	.55	Fixed effects model
Stroke	5/5101	0.85	0.71, 1.02	.09	14%	.33	Fixed effects model
Neurological events	3/2965	1.26	1.02, 1.57	.04	0%	.47	Fixed effects model
TIA	5/5375	1.58	1.14, 2.17	.006	0%	.97	Fixed effects model
MI	4/4718	0.98	0.71, 1.36	.90	0%	.85	Fixed effects model
NOAF	4/3441	0.48	0.38, 0.61	<.00001	68%	.02	Random-effect model
Permanent PM	4/3441	2.61	1.36, 5.00	.004	90%	<.00001	Random-effect model
Rehospitalization	2/3692	1.25	1.06, 1.46	.007	0%	.41	Fixed effects model
Major vascular complications	3/3165	2.38	1.26, 4.49	.007	58%	.09	Random-effect model
Bleeding	3/3165	0.56	0.31, 1.00	.05	96%	<.00001	Random-effect model
All AKI	3/3165	0.63	0.31, 1.30	.21	70%	.04	Random-effect model
Endocarditis	1/2032	1.85	0.69, 4.99	.22			
Reintervention	2/3692	3.22	1.64, 6.29	.0006	0%	.62	Fixed effects model
CAD	1/2032	0.65	0.19, 2.38	.54			

AKI = acute kidney injury, CAD = coronary artery disease, CI = confidence intervals, CV = cardiovascular, NOAF = new-onset atrial fibrillation, RR = risk ratio, TIA = transient ischemic attacks.

### 3.6 Comparison between transcatheter aortic valve implantation and surgical aortic valve replacement regarding to the 5-year outcomes

**Figure 7:**
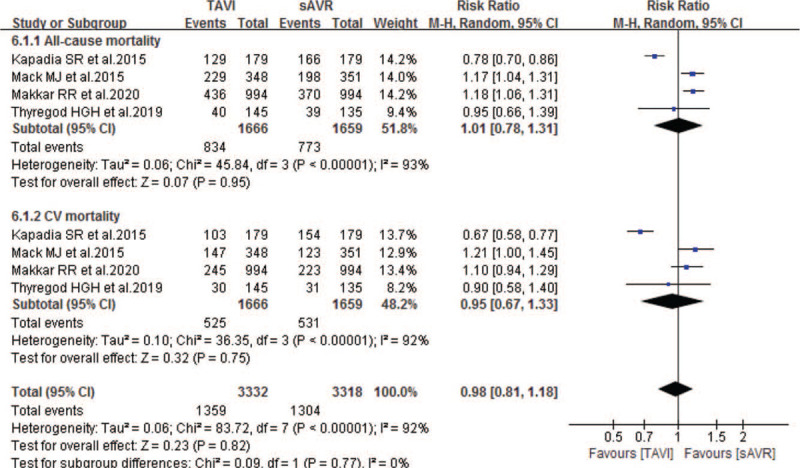
Forest plot of comparison between TAVI and sAVR for severe AS regarding to 5-year mortality. sAVR = surgical aortic valve replacement.

Five studies compared the 5-year mortality between TAVI and sAVR groups. As Fig. 7 showing, our pooled results indicated non-inferiority in the 5-year all-cause and CV mortality of TAVI when compared to sAVR, with pooled RRs of 1.01 (95% CI 0.78–1.31; *P* = .95; 3325 pts) and 0.95 (95% CI 0.67–1.33; *P* = .75; 3325 pts) respectively. Similarly, when compared with sAVR, TAVI showed noninferiority in the following 5-year outcomes: stroke, rehospitalization, MI, endocarditis and permanent pacemaker implantation (Table 6). In addition, one study also showed noninferiority between TAVI and sAVR in 5-year neurological events and renal failure. However, compared with sAVR, TAVI experienced a significantly lower incidence of NOAF (RR 0.46; 95% CI 0.39–0.54; 2268 pts), but higher incidence of TIA (RR 1.50; 95% CI 1.04–2.17; 2967 pts) and reintervention (RR 3.40; 95% CI 1.47–7.85; 2268 pts) respectively (Table 6).

**Table 6:** The pooled results of comparison between TAVI and sAVR for severe AS regarding to the 5-year outcomes.

		Pooled results			Heterogeneity		
Subgroups	No. of study/pts	RR	95% CI	*P* value	*I*^2^	*P*_*h*_ value	Analytical effect model
All-cause mortality	4/3325	1.01	0.78, 1.31	.95	93%	<.00001	Random-effect model
CV mortality	4/3325	0.95	0.67, 1.33	.75	92%	<.00001	Random-effect model
Stroke	4/3325	1.13	0.93, 1.36	.22	0%	.70	Fixed effects model
Rehospitalization	3/3045	0.99	0.52, 1.91	.98	97%	<.00001	Random-effect model
TIA	3/2967	1.50	1.04, 2.17	.03	0%	.88	Fixed effects model
MI	3/2967	1.20	0.90, 1.58	.21	49%	.14	Fixed effects model
Major vascular complications	1/699	2.95	1.64, 5.32	.0003			
Bleeding	1/699	0.73	0.57, 0.95	.02			Fixed effects model
Endocarditis	3/2967	1.40	0.89, 2.20	.14	0%	.64	Fixed effects model
Permanent PM	3/2967	1.94	0.85, 4.40	.11	90%	<.0001	Random-effect model
Neurological events	1/1988	1.24	1.00, 1.53	.05			
NOAF	2/2268	0.46	0.39, 0.54	<.00001	31%	.23	Fixed effects model
Reintervention	2/2268	3.40	1.47, 7.85	.004	0%	.86	Fixed effects model
RF	1/699	1.01	0.58, 1.74	.98			

CI = confidence intervals, CV = cardiovascular, MI = myocardial infarction, NOAF = new-onset atrial fibrillation, RF = renal failure, RR = risk ratio, TIA = transient ischemic attacks.

## 4 Discussion and conclusions

Aortic stenosis is one of the most common valvular problems associated with significant morbidity and mortality in the United States.^[33,34]^ Before TAVI therapy, sAVR was considered the gold standard to improve the prognosis.^[35]^ At present, TAVI has become a valuable therapeutic standard for patients with symptomatic severe aortic stenosis,^[36]^ that was traditionally envisioned to be a treatment option in high-risk surgical candidates.^[37]^ In addition, the encouraging results derived from numerous randomized trials and observational registries corroborate TAVI as a reliable alternative to conventional sAVR in high-risk and intermediate-risk patients and demonstrates a future potential even to moderate to mild risk patients.

At present, several meta-analyzes explored the efficacy of TAVI for patients with symptomatic severe aortic stenosis.^[6,38–45]^ However, there results still failed to reach consensus. For example, the meta-analysis of Polimeni A (2020)^[43]^ with a total of 3 randomized studies showed that TAVI was associated with lower CV mortality compared to sAVR at 1-year follow-up. Nevertheless, paravalvular aortic regurgitation and pacemaker implantation still represent 2 weak spots that should be solved.^[43]^ However, Al-Abdouh A (2020) indicated that there was no difference in all-cause mortality or stroke between TAVI and sAVR, but TAVI was associated with lower risk of other perioperative complications except for moderate-severe paravalvular leak and the need for permanent pacemaker implantation.^[38]^

Thus, the present meta-analysis was designed to comprehensively compare the noninferiority of TAVI with sAVR for patients with severe aortic disease. Our pooled analysis of 14,394 patients showed no difference in 30-day, 1-year, 2-year, and 5-year all-cause or CV mortality as well as stroke between TAVI and sAVR. Regarding to the 30-day outcomes, compared with sAVR, TAVI experienced a significantly lower incidence of MI (RR 0.62; 95% CI 0.40–0.97; 5441 pts), cardiogenic shock (RR 0.34; 95% CI 0.19–0.59; 1936 pts), AKI > stage 2 (RR 0.37; 95% CI 0.25–0.54; 5371 pts), and NOAF (RR 0.29; 95% CI 0.24–0.35; 5371 pts) respectively, but higher incidence of permanent pacemaker implantation (RR 3.16; 95% CI 1.61–6.21; 5441 pts) and major vascular complications (RR 2.22; 95% CI 1.14–4.32; 5371 pts). Regarding to the 1- and 2-year outcomes, compared with sAVR, TAVI experienced a significantly lower incidence of NOAF, but higher incidence of neurological events, TIA, permanent pacemaker implantation and major vascular complications respectively. Regarding to the 5-year outcomes, compared with sAVR, TAVI experienced a significantly lower incidence of NOAF, but higher incidence of TIA and reintervention respectively. From our results, TAVI showed non-inferiority when compared to sAVR in early, mid- or long-term survival. In addition, the incidence of stroke after sAVR and TAVI was equal at 30-day, 1-year, 2-year, and 5-year follow-up. This results was inconsistent with Polimeni A (2020) of 3 randomized studies and nearly 3000 patients which indicated that after 1 year, the risk of CV death was significantly lower with TAVI compared to sAVR (RR = 0.56; 95% CI 0.33–0.95; *P* = .03).^[43]^ Similarly, the author showed no differences between the groups for 1-year all-cause mortality (RR = 0.67; 95% CI 0.42–1.07; *P* = .10) lower risk of NOAF of TAVI compared to sAVR (RR = 0.26; 95% CI 0.17–0.39; *P* < .00001).^[43]^ Adams DH et al (2014) indicated that TAVI with a self-expanding transcatheter aortic-valve bioprosthesis was associated with a significantly higher rate of survival at 1 year than sAVR.^[46]^ In addition, several propensity score-matched analyses showed similar conclusion that TAVI was feasible and comparable to surgery in terms of early, 1-year mortality.^[47–51]^ However, Muneretto et al (2015) suggested that the use of TAVI in patients with an intermediate- to high-risk profile is associated with a higher rate of perioperative complications and decreased survival at the 24-month follow-up compared with the use of conventional surgery or sutureless valves.^[52]^ Kapadia SR (2018)^[53]^ indicated that sAVR was associated with a higher risk of early major stroke than TAVI, which was inconsistent with our pooled results.

Several significant findings should emphasize this analysis which failed to be presented in previous meta-analysis. First, these studies included in this meta-analysis showed good homogeneity and the majority of pooled analyzes were performed using fixed-effect models, which benefited the reliability of results. Second, these RCTs included in this analysis experienced high quality (93.7%) and were about multicenter studies, which strengthened the evidence of the pooled results of this meta-analysis. Third, the present study pooled-analyzed 30 day, 1-year, 2-year, and 5-year outcomes and displayed the dynamic changes of some outcomes. For example, though the incidence of the postoperative permanent pacemaker implantation in TAVI group was significantly higher than sAVR at both 30 day, 1-year and 2-year follow-up, the pooled results finally showed no significant difference of permanent pacemaker implantation between TAVI and sAVR at 5-year follow-up. Fourth, we included 16 studies with 14,394 patients in this analysis and compared 24 groups of characteristics of patients which may influence the outcomes of patients or may result in any risk bias of our results. Our results showed that there was no significant difference between TAVI and sAVR groups in the majority of baseline (Table 2). However, previous studies failed to perform this. Finally, compared with previous analysis, we comprehensively compared the early, midterm and long term clinical outcomes between TAVI and sAVR groups in our analysis, which included 20 30-day outcome indicators (Table 3), 22 1-year outcome indicators (Table 4), 16 2-year outcome indicators (Table 5), and 15 5-year outcome indicators (Table 6).

There existed several limitations in our work. First, due to lack of patient-level data, we could not perform additional subgroup analyses for other baseline characteristics. Though the baseline characteristics were comparable between TAVI and sAVR in included studies, studies have indicated that many population characteristics may influence the postoperative outcomes of patients. For instance, Onorati F (2014)^[54]^ found that female sex was a risk factor for mortality after aortic valve replacement, for major vascular complications after TAVI, and for transfusions after both approaches. Second, there were noticeable variations among the studies with regard to the definition of surgical risk and outcomes, valve type, and delivery system. As most of the studies were done with TAVI devices that are not contemporary, this review is limited to showing the effects of old TAVI devices. Winter MP (2020) conducted an overview on common complications related to the different TAVI devices and demonstrated a gradual improvement in peri-proce-dural mortality and complication with next generation devices as compared with first generation devices.^[55]^ Finally, we did not discuss the medical economics of the index procedures and health benefits measured as the number of added life-years or quality-adjusted life years. It was important to mention that the data regarding cost-effectiveness of TAVI (assessed by incremental cost-effective ratio for life-years or quality-adjusted life years) were more convincing for inoperable or high-risk candidates and predominantly favor affluent countries.

In conclusion, our analysis shows that TAVI was equal to sAVR in early, midterm and long term mortality for patients with severe aortic disease. In addition, TAVI may be favorable in reducing the incidence of both early, midterm and long term NOAF. However, pooled results showed superiority of sAVR in reducing permanent pacemaker implantation, neurological events, TIA, major vascular complications and reintervention. Some outcomes may dynamically change as follow-up time goes on. Thus, future studies should focus on clearing dynamic evolution of mortality and different complications after TAVI or sAVR.

## Author contributions

**Conceptualization:** Yong-Hong Wang, Jian-Ying Wu, Bing Song.

**Data curation:** Rui-Sheng Liu.

**Formal analysis:** Peng-Ying Zhao, Yong-Hong Wang, Bing Song.

**Investigation:** Rui-Sheng Liu, Jian-Ying Wu.

**Methodology:** Peng-Ying Zhao, Yong-Hong Wang, Rui-Sheng Liu, Jian-Ying Wu, Bing Song.

**Software:** Rui-Sheng Liu, Ji-Hai Zhu, Bing Song.

**Writing – original draft:** Peng-Ying Zhao, Yong-Hong Wang, Rui-Sheng Liu, Ji-Hai Zhu, Bing Song.

**Writing – review & editing:** Peng-Ying Zhao, Yong-Hong Wang, Ji-Hai Zhu, Jian-Ying Wu, Bing Song.
